# Increased Responsiveness of Human Coronary Artery Endothelial Cells in Inflammation and Coagulation

**DOI:** 10.1155/2009/146872

**Published:** 2010-01-10

**Authors:** Katja Lakota, Katjusa Mrak-Poljsak, Blaz Rozman, Snezna Sodin-Semrl

**Affiliations:** Department of Rheumatology, University Medical Centre, Vodnikova 62, 1000 Ljubljana, Slovenia

## Abstract

The effects of anti-inflammatory plant extracts, such as black tea extract (BTE) and resveratrol (RSV) could modulate cell activation leading to atherosclerosis, however there is little comparative information about how different endothelial cell types are affected by these compounds. In order to compare human endothelial cells derived from different origins (umbilical vein or HUVEC, coronary artery or HCAEC, microvascular or HMVEC) and their interleukin-1*β* (IL-1*β*) responsiveness, IL-6 ELISA, RT-PCR, tissue factor assay, and prostacyclin responses using 6-keto PGF_1*α*_ ELISA were determined. The IL-1*β*-induced IL-6 levels were dose-dependent with highest responses seen in HCAEC. Significant inhibition of IL-1*β* responses was achieved with BTE and RSV, with the largest decrease of IL-6 and TF seen in HCAEC. Prostacyclin levels were highest in HUVEC and were inhibited by RSV in all cell types. The differences between the endothelial cell types could account for greater susceptibility of coronary arteries to inflammation and atherogenesis.

## 1. Introduction

Coronary artery disease, as an important manifestation of atherosclerosis, is one of the most frequent causes of death and disability in the Western world [1]. It has been proposed that inflammation is the driving force in atherosclerosis [2, 3], and strong evidence supports the central role of proinflammatory cytokines, such as interleukin-1*β* (IL-1*β*) and interleukin-6 (IL-6) in these pathologies. Human coronary artery endothelial cells (HCAEC) have previously been shown to have a strikingly greater responsiveness than human umbilical vein endothelial cells (HUVEC) [4].

The endothelium is considered a dynamic organ with secretory, metabolic, immunologic roles, in addition to its regulatory function of nutrient transport. Endothelial cells from diverse environments are heterogenous with respect to their surface phenotype, mRNA expression, and levels of IL-6 and procoagulant proteins, such as tissue factor (TF). Within the past decades, the majority of endothelial proinflammatory/coagulation studies have been performed on HUVEC, derived from a vascular bed not present in the adult, making these cells an inappropriate model of endothelial inflammation and coagulation [5, 6].

TF is a pivotal factor found associated with endothelial cells within atherosclerotic plaques [7] that can transform the endothelial cell membrane from an anticoagulant to a procoagulant surface following vascular injury [8]. Unique sensitivity of HCAEC to tumor necrosis factor *α* (TNF*α*)-stimulated expression of adhesion molecules (as compared to human aortic endothelial cells) [9] and greater susceptibility of HCAEC to inflammatory stimuli, (as compared to HUVEC and human dermal microvascular endothelial cells (HMVEC)) [4] have been reported. Endothelial cells contribute to homeostasis and vasodilation, also by releasing prostacyclin (PGI_2_) known to inhibit platelet aggregation and deposition [5]. However, no reports as far as we know, have addressed the comparative influence of IL-1*β* on PGI_2_ release, TF activity, or IL-6 protein release from HCAEC versus other endothelial cell types. 

Previous epidemiological studies have suggested that black and green tea consumption may have beneficial effects on endothelial function and is associated with a decreased risk of cardiovascular events [10–13]. Black tea inhibited the proliferation of smooth muscle cells involved in the development and progression of atherosclerosis [14] and mechanisms for the beneficial effects of tea including vasculoprotective, antioxidative, antithrombogenic, anti-inflammatory, and lipid-lowering properties of tea flavonoids have been reported [11, 15–18]. Animal studies confirmed that black tea extract (BTE) has anti-inflammatory activity [15], however, its effects on different types of endothelial cells is still not clear. 

The polyphenolic stilbene resveratrol (RSV) is found in grape skins, red wine, and peanuts [19, 20]. Trans -RSV has been shown to inhibit TF expression in vascular cells and to act anti-inflammatory [21–23]. RSV also attenuated TNF*α*-activated HCAEC through the NF-kB pathway [24]. However, it is still largely unknown how BTE and/or RSV affect IL-1*β*-stimulated inflammatory and coagulation pathways in HCAEC, as a novel model system, compared to HUVEC or other endothelial cell types.

So, our focus has been to elucidate the mechanisms that could modulate IL-1*β* proinflammatory/procoagulant responses in HCAEC. The specific objectives of this study were to understand the influence of potential anti-inflammatory plant extracts, such as BTE and RSV, on IL-1*β*-induced primary HCAEC and to compare their IL-6, TF, and prostacyclin responses with other types of endothelial cells.

## 2. Materials and Methods

### 2.1. Materials

Lyophilized human IL-1*β* (Invitrogen - Carlsbad, California, USA), resveratrol and extract from black tea (Sigma - Saint Louis, Missouri, USA) were reconstituted according to manufacturer's instructions to stock concentration and stored until usage at −20°C or −80°C. The black tea extract used in the current report is composed of more than 80% theaflavins (theaflavin and theaflavin gallates). The final concentration of IL-1*β* was 1000 pg/mL, resveratrol 40 *μ*mol/L, and the final concentration of black tea extract was 40 *μ*g/mL unless otherwise stated.

### 2.2. Cell Culture

HUVEC, HCAEC, and HMVEC were purchased from Cambrex BioScience (Walkesville, Maryland, USA). The cells were plated onto 12 or 6 well plates or 75 cm^2^ flasks (TPP, Trasadigen, Switzerland) at 37°C in a humidified atmosphere at 5% CO_2_. HCAEC and HMVEC were grown in EGM-2M medium (Cambrex BioScience, Walkesville, Maryland, USA) containing 5% fetal bovine serum; for HUVEC we used EGM medium containing 2% fetal bovine serum (Cambrex BioScience, Walkesville, Maryland, USA). For experiments, subconfluent cell cultures were used between 4 and 6 passages in serum-free medium with addition of stimulatory and/or modulatory substances for 24 hours, unless otherwise indicated. Prior to experiments, cells were incubated in serum-free media for 2 hours.

### 2.3. Measurement of Secreted Interleukin-6 and Prostacyclin Metabolite 6-Keto PGF_1*α*_

IL-6 from cell culture supernatants was measured and analyzed by human IL-6 ELISA from BioSource International (Camarillo, California, USA) according to manufacturer's instructions. Enzyme immunoassay (EIA) 6-keto PGF_1*α*_ (Cayman diagnostica, Michigan, USA) was used to measure the concentration of nonenzymatically converted metabolite of prostacyclin or PGI_2_ in cell culture supernatants with competitive EIA according to the instructions of the manufacturer. 6-keto PGF_1*α*_ is a stable product of PGI_2_.

### 2.4. RNA Isolation and Reverse Transcription Polymerase Chain Reaction (RT-PCR) Analysis

Before RT-PCR, total RNA from endothelial cell cultures was isolated using Total RNA Isolation System (Promega, USA) following manufacturer's instructions. The purity and amount of RNA were determined by measuring the OD at a ratio of 260 to 280 nm. 1 *μ*g of total RNA were transcribed into DNA by Reverse Transcription System (Promega, USA) and PCR was performed for *β*-actin, IL-6, and TF (Table 1). *β*-actin was used as a control for normalization. 

### 2.5. Tissue Factor Activity Assay

Tissue factor activity was measured following the Actichrome TF (American diagnostica, Stamford, Connecticut, USA) procedure. After cell treatment in 12 well plates, the medium was removed and 150 *μ*L/well of TBS/Triton X-100 buffer was added. Cells were then scraped and frozen at −80°C for 15 minutes and then thawed at 37°C. This freeze-thaw cycle was repeated twice. Subsequently, cells were kept at 37°C for another half an hour. Cell lysates were then mixed with Factor VIIa and Factor X in 96 well plates. Following a 15-minute incubation at 37°C, Spectrozyme FXa was added for 20 minutes and the reaction was stopped with glacial acetic acid. The absorbance at 405 nm was read on the Tecan Sunrise Colorimeter. The standard curve was constructed from provided standards and corresponding sample concentrations were calculated.

### 2.6. Statistical Analysis

All experiments were repeated three times and the mRNA expression studies were shown as 1 representative gel of two performed. Data are presented as a mean ± standard deviation (SD). Means were compared between the treated and control groups using Student's *t*-test. *P* values less than .05 were determined to be statistically significant.

## 3. Results

**Figure 1:**
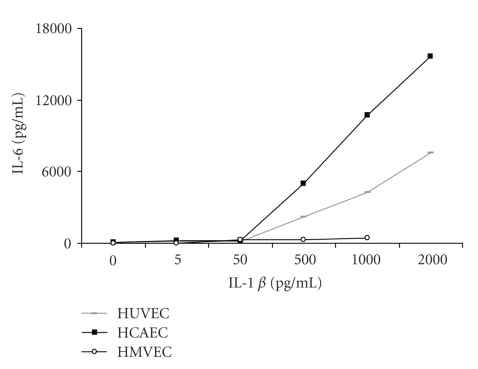
Dose dependent responses of HUVEC, HCAEC, and HMVEC to IL-1*β* (0–2000 pg/mL) as measured by ELISA of released IL-6 protein levels from cell supernatants. Results are expressed as a mean of three separate experiments.

In order to determine the inflammatory/coagulation responses of primary human endothelial cells, in particular HCAEC, stimulated with different doses of IL-1*β*, we measured released IL-6 protein (Figure 1). Semiconfluent primary HUVEC, HCAEC, and HMVEC were incubated in 6-well plates with increasing doses of IL-1*β* (from 0 to 2000 pg/mL) for 24 hours in serum-free media. Released protein levels of IL-6 were measured in supernatants by ELISA, and in parallel, mRNA expressions of *β*-actin, IL-6, and TF were determined using RT-PCR. A comparison of released IL-6 protein levels in the endothelial cell types showed highest levels coming from HCAEC (approximately 2-fold higher than from HUVEC) and the lowest from HMVEC (Figure 1). The first rapidly increased IL-6 protein levels were seen at an IL-1*β* concentration of 500 pg/mL in HCAEC and HUVEC, in comparison to HMVEC where responses were consistently low. The mRNA expression results show that IL-6, as well as TF mRNA expression dose dependently increased with IL-1*β* in all cell types (data not shown).

**Figure 2:**
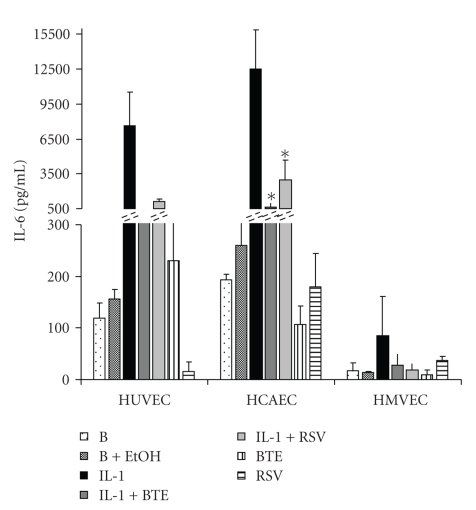
Released IL-6 protein levels as measured by ELISA of supernatants from HUVEC, HCAEC, and HMVEC incubated with IL-1*β* (1000 pg/mL) for 24 hours in the absence or presence of BTE (40 *μ*g/mL) and RSV (40 *μ*M). Data represent the mean ± SD of three separate experiments. *P < .05 compared with IL-1*β* levels of expression.

**Figure 3:**
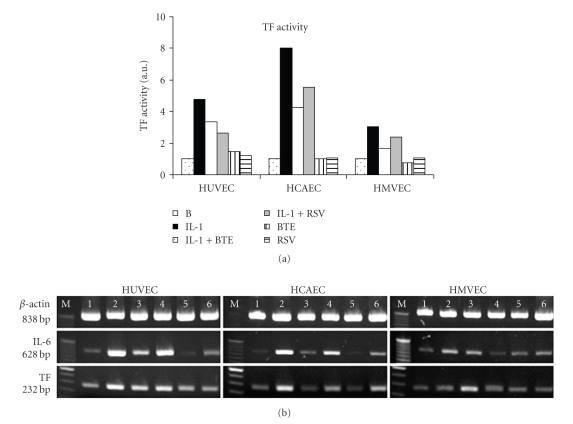
(a) TF activity was measured in the indicated treatments. Data are presented as arbitrary units, which indicate fold-change above background TF levels and were generated from the mean of three separate experiments. (b) mRNA expressions of *β*-actin, IL-6, and TF are shown from HUVEC, HCAEC, and HMVEC incubated with IL-1*β* (1000 pg/mL) in the absence or presence of BTE (40 *μ*g/mL) and RSV (40 *μ*M). The treatments of cell cultures were background control (lane 1), IL-1*β* 1000 pg/mL (lane 2), IL-1*β* + BTE (lane 3), IL-1*β* + RSV (lane 4), BTE (lane 5), and RSV (lane 6). Results shown are from one representative experiment of two separate ones performed.

To investigate the effects of modulatory molecules on IL-1*β*-stimulated endothelial cells, we measured the IL-6 protein levels in the presence or absence of BTE and RSV. Both BTE and RSV significantly inhibited IL-6 protein levels of HCAEC (Figure 2). IL-1*β*-induced TF activity showed the highest response in HCAEC and was inhibited by BTE and RSV in all three endothelial cell types (Figure 3(a)). The IL-1*β*-induced mRNA expression of IL-6 and TF in all endothelial cell types is down-regulated with BTE and RSV (Figure 3(b)).

**Figure 4:**
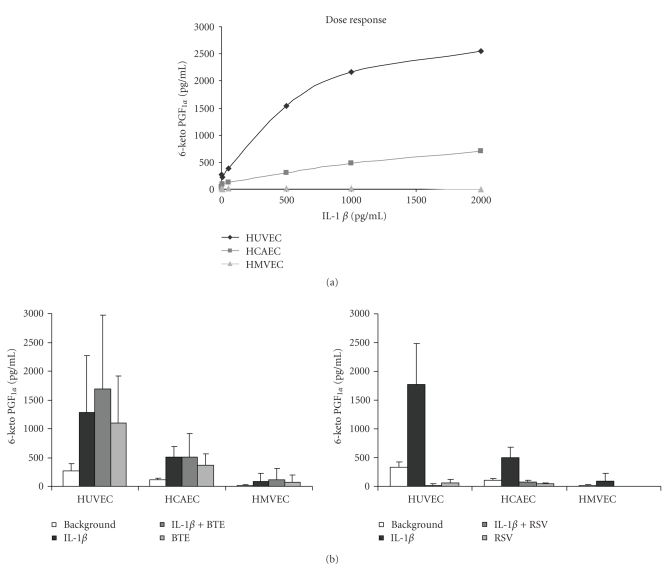
(a) IL-1*β* dose-dependent prostacyclin release, as measured by 6 keto-PGF_1*α*_, from HUVEC, HCAEC, and HMVEC. Concentrations of IL-1*β* were 0–2000 pg/mL. Data represent the mean of three separate experiments. (b) PGF_1*α*_ levels measured in the indicated endothelial cell types treated with IL-1*β* (1000 pg/mL) and/or BTE (40 *μ*g/mL) [left panel] or IL-1*β* (1000 pg/mL) and/or RSV (40 *μ*M) [right panel]. Data represent the mean ± SD of three separate experiments.

To investigate the effects of IL-1*β* on homeostasis of endothelial cells, released prostacyclin PGI_2_ was measured as 6-keto PGF_1*α*_ in cell supernatants (Figure 4). To determine whether the responses were dose dependent, HCAEC, HUVEC, and HMVEC were incubated with increasing concentrations of IL-1*β* (Figure 4(a)). The highest responses were shown in HUVEC, which were around 3-4 fold higher than in HCAEC. PGF_1*α*_ levels from IL-1*β*-stimulated HMVEC were below the detection limit. Contrary to IL-6 levels and TF activity, BTE addition did not cause inhibition of IL-1*β*-stimulated prostacyclin release (Figure 4(b), left panel). However, RSV abrogated IL-1*β*-induced 6-keto PGF_1*α*_ levels in all three cell types (Figure 4(b), right panel). 

## 4. Discussion

In this report, HCAEC were used as a model cell system, shown to be highly responsive to IL-1*β*-stimulated IL-6 released protein levels, TF activity, and inhibition of these by BTE and RSV. Studies on HCAEC reported in literature have looked at different stimulating molecules, such as IL-1*α* and TNF*α*, activated protein C, lipopolysaccharide [25–28], and their effects on different cytokine and chemokine expression, indicating unique cell type patterns and/or levels of expression. 

Lakota et al. previously indicated that HCAEC display greater susceptibility to inflammation and potential atherogenesis than HUVEC or HMVEC [4]. This is in accord with IL-1*β*-stimulated HCAEC data indicated in this report. Among the most accessible natural substances used worldwide are black tea, green tea, and wine, which have been suggested to be important modulators of cardiovascular disease (CVD). Both black and green tea have shown acute beneficial effects on aortic stiffness and wave reflections, as reported by Vlachopoulos et al. [29]. Since aortic stiffness and wave reflections are markers of CVD and prognostic factors of cardiovascular risk, they are important parameters to be studied in healthy individuals [29]. Many epidemiological studies of flavonoid consumption however have been performed with mixed results. One possible explanation for the lack of cardiovascular protection in some of the studies is the difference in infusion time, stirring, leaf size, and measurements in plasma/whole blood changes, although different brands and addition of milk did not show significant variances [30]. However, flavonoids were reported to bind to protein, which causes variations in bioavailability and also results in changed biological activity especially in the case of binding to specific receptors and/or enzymes [31, 32]. However, overall, the evidence suggests that individuals with the highest flavonoid intake have modestly reduced risks for CVD (as reviewed in Vita JA, 2005 [33]). For tea, this conclusion is supported by a meta-analysis with 10 cohort studies and seven case-control studies included, which suggested an overall reduction in CVD risk of around 11% with consumption of 3 cups of tea per day [34]. A meta-analysis was also performed recently on the association between green or black tea consumption and the risk of stroke [35]. Data from 9 studies involving 4 378 strokes among 194 965 individuals were pooled. The authors conclude that although a randomized clinical trial would be necessary to confirm the effect, this meta-analysis suggests that regardless of their country of origin, individuals consuming 3 cups of tea per day had a 21% lower risk of ischemic stroke than those consuming less than 1 cup per day. In a large sample (6 597 subjects) of the elderly (over 65 years), Debette et al. reported for the first time that carotid plaques were less frequent with increasing tea consumption in women [36]. 

Usually 1 g of tea is used for 100 mL of infusion [30, 32]. In the process of manufacturing, the black tea leaf catechins are allowed to oxidize into theaflavins which give the black tea its characteristic colour and taste. The black tea flavonoid content accounts for 20%–30% catechins, 10% theaflavins and 50%–60% thearubigins [37], and both catechins as well as theaflavins have been shown to act as cardio-protectants in cardiomyocytes [38]. Thearubigins are poorly characterised and the bioavailability of theaflavins is poorly understood [37].

The present data in HUVEC, HCAEC, and HMVEC indicate that BTE significantly abrogates IL-1*β*-induced IL-6 protein released levels similarly to RSV (Figure 2), while also decreasing both TF activity levels and expression (Figure 3). However, BTE does not seem to have any effects on IL-1*β*-induced PGI_2_ in any of the three cell types (Figure 4(b), left panel). These differences imply that cytokine-specific processes and differential signaling pathways might be involved. Since black tea consumption has been previously associated with a decreased risk of cardiovascular events [10, 11], signaling events in response to BTE are relevant. Bovine aortic endothelial cells when exposed to BTE showed eNOS activity mediated through p38 MAPK and estrogen receptors leading to phosphatidylinositol 3-kinase/Akt pathway and eNOS generation [39, 40] and vasorelaxation of aortic rat rings [40].

RSV-mediated cardio-protection is achieved through the preconditioning effect and thus achieves a number of cardio-protective functions (reviewed in [19]), such as effecting release and/or generation of inflammatory mediators and attenuation of various soluble intercellular cytokines. RSV functions in scavenging free radicals and inhibiting lipid peroxidation, up-regulation of inducible NO synthase, vascular endothelial growth factor, kinase insert domain-containing receptor, and endothelial NO synthase. Adenosine receptors also have an important function in the RSV preconditioning.

Dealcoholized wine (1 cup) delivered in a single oral dose to healthy subjects less than 40 years old was found to increase endothelium-dependent vasodilation [41]. The authors indicate that this adds support to the hypothesis that antioxidant properties of red wine, rather than ethanol, may protect against cardiovascular diseases, however more research is needed on subjects with coronary heart disease. Usually the concentrations of RSV in cellular models of CVD protection are 0.1–100 *μ*mol/ L [42], although some studies showed different effects in low/high doses in enhancing proliferation and inducing apoptosis [43].

When RSV in humans is absorbed around 75% is excreted via feces and urine. Serum levels were independent from meals and its lipid content [44], but RSV is rapidly metabolized into glucuronides and sulfates [20], which stay in the blood for 9 hours [45]. Biologic activity of metabolites was not elucidated yet [20, 42], and it is suggested that prolonged administration could lead to increased concentrations [42, 46]. It is necessary to apply caution when interpreting the literature data translating concentrations of RSV on potential cardiovascular effects.

To our knowledge, this is the first report to show the effects of RSV on IL-1*β*-induced IL-6 and TF responses in HCAEC, which could serve in cardioprotective processes similar to the ones described previously [24, 42, 47, 48]. RSV has been shown to inhibit TF (at 5–100 *μ*mol/L) in HUVEC [21] at comparable concentrations to the 40 *μ*M used in the present report, to reduce expression of adhesion molecules on stimulated human saphenus vein endothelial cells [49], to inhibit adhesion of activated platelets to collagen or fibrinogen [50] and lower ICAM, VCAM [24, 51]. RSV was also reported to enhance the inhibitory activity of PGI_2_ on platelet aggregation in low doses [52], as well as to inhibit TNF*α*-induced NAD(P)H oxidase and NF-*κ*B activation and inflammatory markers (at 0.1–10 *μ*mol/L) [24, 42, 51], similarly to our results. In porcine coronary arteries, short term treatment with RSV significantly inhibited MAPK activities, with reduced phosphorylation seen of ERK1/2, JNK-1 and p38 MAPK [53, 54], and STAT3 phosphorylation [55]. RSV was also found to inhibit protein kinase C in 2 *μ*M concentration [56].

The data shown in HUVEC (Figure 4) indicates a similar level of IL-1*β*-stimulated PGI_2_ (measured using 6-keto PGF_1_ *α*) as shown by Olszanecki et al. [57]. All three cell types HUVEC, HCAEC, and HMVEC indicate a similar vasoregulatory role for IL-1*β*, while RSV addition abrogated PGI_2_ levels (Figure 4(b), right panel).

RSV has also been shown to decrease the expression of vasoconstrictor endothelin and increase eNOS in HUVEC, which might counterbalance PGI_2_ inhibition [58] and decrease NAD(P)H oxidase activity [59].

In conclusion, a growing body of evidence indicates that inflammation not only provides the baseline for future atherosclerotic events, but is a necessity for coronary plaque formation and coagulation leading to thrombosis. The unique responsiveness of HCAEC could account for the greater susceptibility of coronary arteries to inflammation and atherogenesis leading to cardiovascular pathology and substances, such as BTE and RSV, also influence these effects at the cellular level.

## Figures and Tables

**Table 1:** Primer sequences and PCR conditions.

	Primer sequences	Denat. *t*/*T*	Anneal. *t*/*T*	Extens. *t*/*T*	No. of cycles
*β*-actin	sense 5′-ATC TGG CAC CAC ACC TTC TAC AAT GAG CTG CG–3′	1 min/94°C	1 min/59°C	1.5 min/72°C	25
	antisense 5′-CGT CAT ACT CCT GCT TGC TGA TCC ACA TCT GC–3′				

IL-6	sense 5′-ATG AAC TCC TTC TCC ACA AGC GC-3′	1 min/94°C	1 min/51°C	1.5 min/72°C	30
	antisense 5′-GAA GAG CCC TCA GGC TGG ACT G-3′				

TF	sense 5′-ACT ACT GTT TCA GTG TTC AAG CAG TGA TTC-3′	1 min/94°C	1 min/52°C	1.5 min/72°C	35
	antisense 5′-ATT CAG TGG GGA GTT CTC CTT CCC AGC TCTG-3′				

*t*/*T*: time and temperature, Denat.: denaturation, Anneal.: annealing, Extens.: extension.
